# Pixel-by-Pixel Arterial Spin Labeling Blood Flow Pattern Variation Analysis for Discrimination of Rheumatoid Synovitis: A Pilot Study

**DOI:** 10.2463/mrms.tn.2015-0145

**Published:** 2016-05-06

**Authors:** Taro Sakashita, Tamotsu Kamishima, Hiroyuki Sugimori, Minghui Tang, Atsushi Noguchi, Michihito Kono, Kenneth Sutherland, Tatsuya Atsumi

**Affiliations:** 1 Graduate School of Health Sciences, Hokkaido University; 2 Faculty of Health Sciences, Hokkaido University, N12-W5, Kita-ku, Sapporo Hokkaido 060-0812, Japan; 3 Department of Radiology, Hokkaido University Hospital; 4 Internal Medicine 2, Hokkaido University Hospital; 5 Graduate School of Medicine, Hokkaido University

**Keywords:** ASL, hand, post labeling delay, rheumatoid arthritis

## Abstract

We examined the capability of a gray-scale arterial spin labeling blood flow pattern variation (BFPV) map with two different post labeling delay (PLD) times to discriminate pannus in patients with rheumatoid arthritis (RA) at 3T. There was a statistically significant difference in the BFPV values between artery, pannus, and surrounding tissue. Furthermore, the color-coded BFPV map was able to accurately distinguish pannus from other tissues. These results suggest this approach may be capable of identifying pannus noninvasively.

## Introduction

An arterial spin labeling (ASL) perfusion map, which is calculated by the subtraction of an image without blood preparation from that with blood preparation, is capable of quantitatively measuring microvascular perfusion characteristics of tissues without the need to administer contrast agents. It may visualize hyperemia of inflamed synovitis (pannus) of rheumatoid arthritis (RA)^1^ because an increased number of capillaries is observed in the inflamed synovial membrane as compared to the normal synovial membrane.^2,3^ However, it has a similar difficulty as conventional contrast enhanced magnetic resonance (MR) imaging of distinguishing pannus formation from the peripheral artery due to their resemblance in signal intensity. This is because both pannus and the peripheral artery have abundant blood flow. Previous studies have attempted to extract microvascular flow from pannus by fixing the upper limit value of the synovial tissue on the ASL perfusion map.^1^ However, this method may be unreliable because perfusion values are dependent on delay time (inversion time for a flow sensitive alternating inversion recovery (FIAR) sequence and post-labeling delay (PLD) for pulsed continuous ASL (pCASL)) to allow the label to reach the tissue of interest.^4^

To overcome the aforementioned disadvantages of ASL perfusion imaging techniques, we introduce the gray-scale/color-coded blood flow pattern variation (BFPV) maps, which represent PLD time-independent pixel-by-pixel relative variation in blood flow pattern. We hypothesize that these maps may help to identify pannus formation by detecting characteristics of angiogenic tissues related to the temporal changes of the labeled blood flows or BFPV. This study aims to test the capability of the gray-scale/color-coded BFPV maps to distinguish pannus from other tissues.

## Methods

### Patients

Eight subjects (7 women and 1 man) with RA of the wrist or finger joints participated in the study. The geometric mean of the age was 57 years with a range from 38 to 67 years. All patients were diagnosed with RA according to the American College of Rheumatology (1987) criteria.^5^ The inclusion criteria of this arthritis cohort consisted of active arthritis, based on clinical findings, of at least the wrist, or finger joint, but without the knowledge of the extent of the disease, with disease duration of less than 1 year. All patients managed in a dedicated biological therapy clinic in a universal hospital were assessed for continuation on biological treatments and reasons for switching to an alternative biological agent or cessation of treatment. All subjects were recruited from consecutive patients admitted to a university hospital. Ethics board permission and written informed patient consent were obtained for this study.

### MR imaging

Images were acquired with a 3.0-T MR imager (DISCOVERY MR750w, GE MEDICAL SYSTEMS, Milwaukee, WI, USA). A 16-channel GE musculo-skeletal flex small coil was applied for signal reception. The patients were placed in the prone position with the arm extended over the head. The hand and the wrist were tightly fixed with sand bags. In all subjects, the hand and the wrist of the dominant side with the joint of the strongest clinical symptoms was examined.

**Fig 1.**
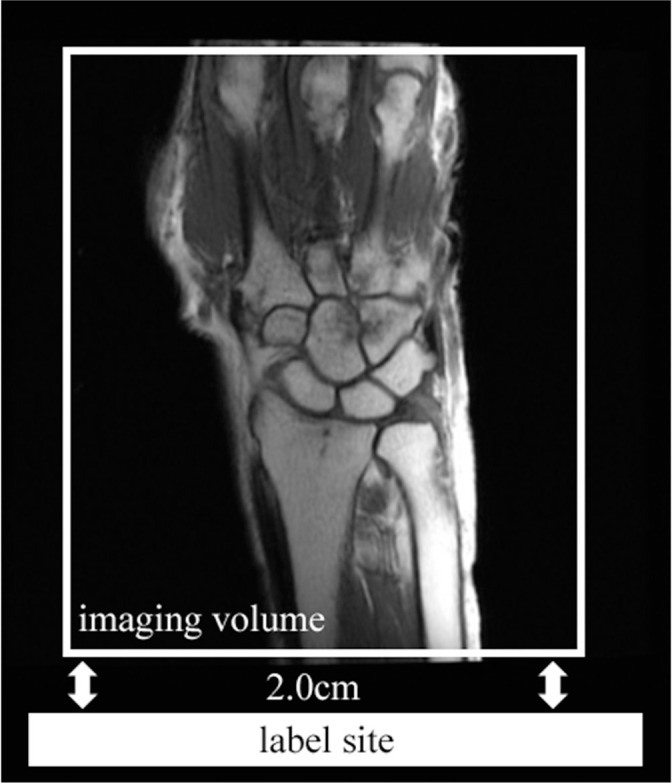
The labeling site utilized in this study.

Three-dimensional pulsed continuous ASL (pCASL) sequences (repetition time/echo time 4397–4852/10.704 ms, slice thickness 4 mm, matrix size 128 × 128, field of view 240 × 240 mm, number of slices 36) for quantitative assessment of tissue perfusion was used. The labeling duration was 1450 ms. As shown in Fig. 1, labeling site was located at 2.0 cm proximal from the end of the imaging volume to avoid imperfect matching of direct off-resonance irradiation effects on the tissue signal for label and control.^6^ Multi-shot spiral 3D fast spin-echo readout was achieved, started after PLD times of 1025, 1525, 2025 ms after the inversion pulse.

Three-dimensional contrast-enhanced liver acquisition with volume acceleration (LAVA) dynamic sequence (repetition time/echo time 7.769–9.114/2.328–2.604 ms, slice thickness 2.0 mm, matrix size 360 × 360, field of view 160 × 160 mm, bandwidth 244.141 Hz/pixel, flip angle 12, acquisition time per phase 13.27 s, number of slices 40–52, phase 26) was used for determining the location of synovitis. A bolus of a contrast agent (0.2 ml/kg of body weight of gadopentetate dimeglumine (Magnevist, Bayer HealthCare, Osaka, Japan) followed by a 20 ml saline chase was delivered at an injection rate of 2 ml/s by using an automatic injection device (Sonic Shot, Nemoto Kyorindo co. Ltd. Tokyo, Japan).

### MR imaging data analysis

The quantitative pCASL (pseudo-continuous arterial spin labeling) perfusion maps were computed on a pixel-by-pixel basis from the analysis of the magnetization difference Δ*M* using the eqn. 1^7–9^:

(1) $$f=6000\frac{\Delta M\;\text{exp}\left(\frac{w}{T_{1\text{a}}}\right)\left(\frac{TE}{T_{2\text{a}}^{*}}\right)}{\rho M_{0\text{a}}2\alpha T_{1\text{a}}}$$

*f*, the perfusion in ml per 100 g/min; *w*, post labeling delay; *T*_1a_, the longitudinal relaxation time of arterial blood (1.8 s); *TE*, the echo time of the sequence; $T_{2\text{a}}^{*}$ , the transverse relaxation time of the arterial blood (50 ms); *ρ*, the density of brain tissue (1.05 g/ml) [21]; *M*_0a_, the equilibrium magnetization of the arterial blood; and *α*, the labeling efficiency (0.85).

The gray-scale BFPV maps were calculated using the following model on a pixel-by-pixel basis using MATLAB software (MathWorks, Natick, MA, USA):

(2) $$BFPV=\frac{rf_{2}-rf_{1}}{w_{2}-w_{1}}$$

*w*_1_, post-labeling delay (1025 ms); *w*_2_, post-labeling delay (2025 ms); *rf*_1_, relative perfusion value with PLD time set 1025 ms; *rf*_2_, relative perfusion value with PLD time set 2025 ms.

### Region of interest selection

**Fig 2.**
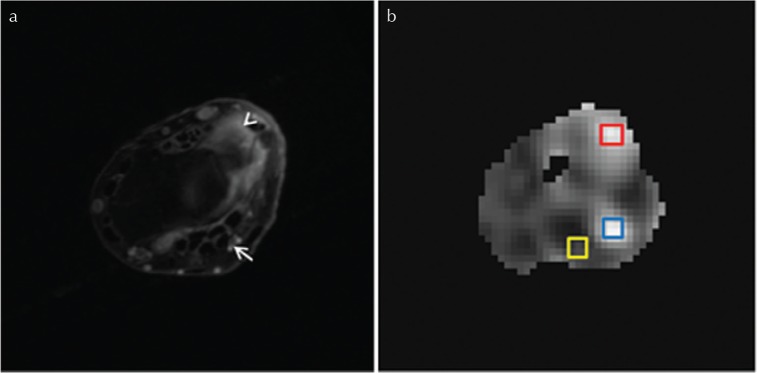
ROIs for measurement of the BFPV and perfusion values. (**a**) The location of the peripheral artery (arrow), pannus (arrow head), and surrounding tissue were determined on post-gadolinium-DTPA images. (**b**) ROIs of the peripheral artery (blue box) and pannus (red box) and surrounding tissue (yellow box) were set and measured on the gray-scale BFPV maps and the pCASL perfusion maps. ROIs, region of interests; ASL, arterial spin labeling; BFPV, blood flow pattern variation; DTPA, diethylene triamine pentaacetic acid; pCASL, pseudo-continuous arterial spin labeling.

From the images of eight patients obtained at 3 min after contrast administration, 87 images in five patients were found to be visualized with artery and pannus on the same image. In each image, the location of the radial or ulnar artery, pannus, and surrounding tissue were determined on the basis of visual analysis of pre- and post-contrast images. In the study, surrounding tissue was defined as a soft tissue except synovial membranes and angiogenic tissues. We anticipated that the signal from labeled arterial blood would fluctuate depending upon the distance of slice level from the labeled plane. Therefore, we evaluated the artery and pannus on the same image so that we can avoid the effect of the signal fluctuation. We also took a special care to adjust the number of the ROI for each tissue type in a given slice. Rectangular regions of interests (ROIs) were placed inside these three different structures so that the size of the ROIs was maximum when the ROIs were within the boundary of these structures (Fig. 2). Images were assessed by a musculoskeletal radiologist with more than 15 years of experience. Subsequently, BFPV and perfusion values of each tissue in the ROI were measured on the gray-scale BFPV maps and the pCASL perfusion maps, respectively, created using MATALB software and ImageJ (National Institutes of Health, Bethesda, MD, USA, http://rsbweb.nih.gov/ij/).

According to the theoretical background of the dynamic tissue perfusion pattern, BFPV values should be high in the order of artery, pannus, and the other tissue. Together with the preliminary study results, we categorized the BFPV values as a color-coded BFPV map. Color-coded BFPV maps were created for all images by setting BFPV threshold values defined as follows: BFPV values of the artery of 25 and more of the interquartile range were defined as artery voxels. BFPV values of pannus of 25–75 of the interquartile range were defined as pannus voxels, and the others defined as surrounding tissue voxels. In addition, the pannus area of each slice, which is located from the distal radio-ulnar joint to the carpometacarpal joints, was measured. The total volumes of pannus (*Pan*_thre_) were calculated by the summation of the slices using the following formula:

(3) $$Pan_{\text{thre}}=\text{number}\;\text{of}\;\text{voxels}\times \text{pixel}\;\text{size}\times \text{slice}\;\text{thickness}$$

On the other hand, manual segmentation was done on post-gadolinium-DTPA images using EV Insite (PSP Corporation, Tokyo, Japan). The pannus formation located from the distal radio-ulnar joint to the carpometacarpal joints of each MRI slice was outlined manually, using a computer mouse, and the areas were calculated automatically. The total volume of pannus (*Pan*_man_) was calculated by the summation of slices using the following formula:

(4) $$Pan_{+}=\sum (\text{the}\;\text{area}\;\text{of}\;\text{pannus}\times \text{slice}\;\text{thickness})$$

Patients were classified into two groups depending on the degree of *Pan*_man_; patients who showed apparent pannus formation were categorized as high-activity group, while patients who showed no or a limited extent of hyperemia in pannus were categorized as low-activity group. Detailed manner of discrimination in activity is described in the results section.

### Statistical analysis

Standard software (PASW Statistics ver. 18.0, IBM Co., Armonk, NY, USA) was used for statistical analysis. A nonparametric Tukey test was used to compare the values of each tissue in the ROI on gray-scale BFPV maps and pCASL perfusion maps.

The independent *t*-test was used to analyze differences of *Pan*_thre_ between high-activity group and low-activity group. A *P-*value <0.05 was considered to indicate a statistically significant difference.

## Results

**Fig 3.**
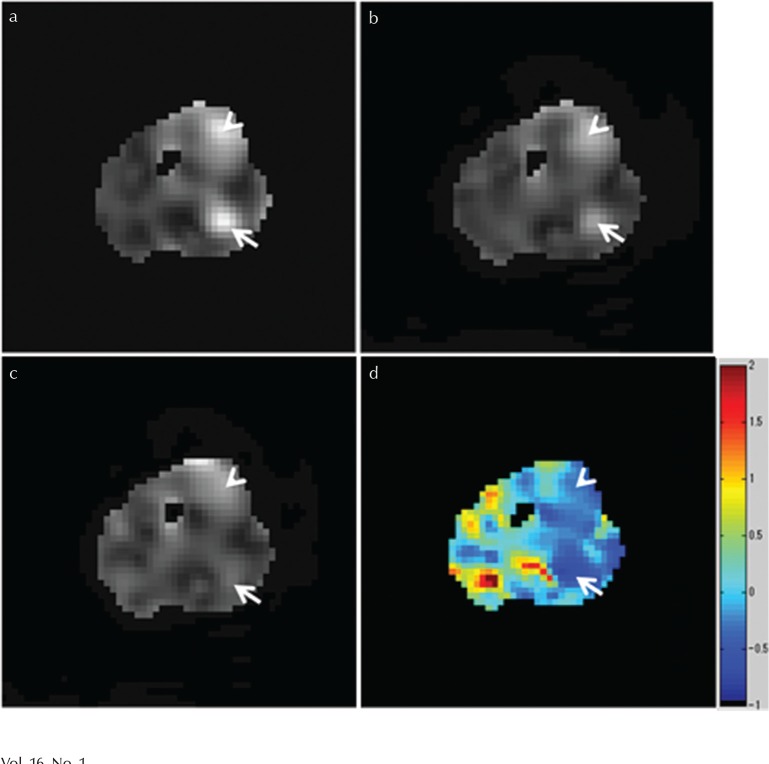
Axial MR images of a RA wrist. pCASL perfusion maps with PLD times of (**a**) 1025, (**b**) 1525, (**c**) 2025 ms are calculated by subtracting an image without blood preparation from that with blood preparation. (**d**) The BFPV map is presented as a color-coded image according to the BFPV values. The BFPV values of the radial artery (arrow) shows negative and smaller than those of pannus (arrow head). RA, rheumatoid arthritis; pCASL, pseudo-continuous arterial spin labeling, PLD, post-labeling delay, BFPV, blood-flow pattern variation.

**Fig 4.**
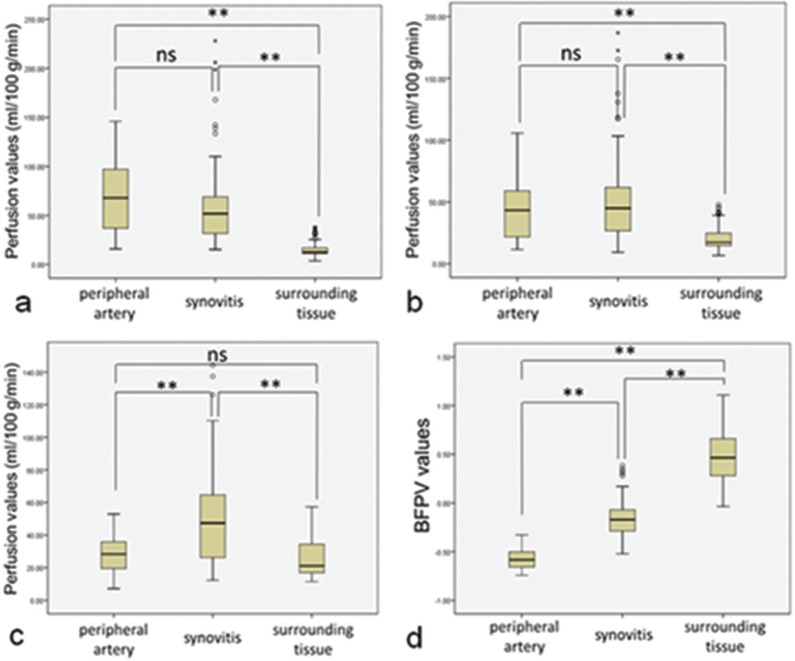
Comparison of ASL perfusion and BFPV values for artery, pannus, and surrounding tissue. Data (average ± standardized deviation) derived from pCASL perfusion maps with PLD times of 1025 (**a**), 1525 (**b**), 2025 (**c**) ms, and the color-coded BFPV map (**d**) are demonstrated. ASL, arterial spin labeling; BFPV, blood flow pattern variation; pCASL, pseudo-continuous arterial spin labeling; PLD, post labeling delay; **, *P* < 10^−8^; ns, not significant.

All images of gray-scale/color-coded BFPV and pCASL perfusion maps were successfully calculated. A typical gray-scale BFPV map is shown in Fig. 3 in comparison to pCASL perfusion maps of the same slice. Five of eight patients showed apparent pannus formation on ASL imaging that corresponded with visual findings of active RA on dynamic imaging. Three patients showed a limited extent of hyperemia of pannus on ASL imaging and dynamic imaging. The Tukey test was performed on 87 images which contained both artery and pannus among 288 images. The comparison of perfusion values of each tissue at different PLDs and BFPV values are shown in Fig. 4. In the pCASL perfusion maps with PLD times of 1025, 1525, 2025 ms, the mean perfusion value revealed a statistically significant difference between two of three combinations of artery, pannus, and surrounding tissue, but didn’t show a statistically significant difference at the same time. On the other hand, there was a statistically significant difference in the BFPV values between these tissues at the same time (*P* < 10^−8^).

**Fig 5.**
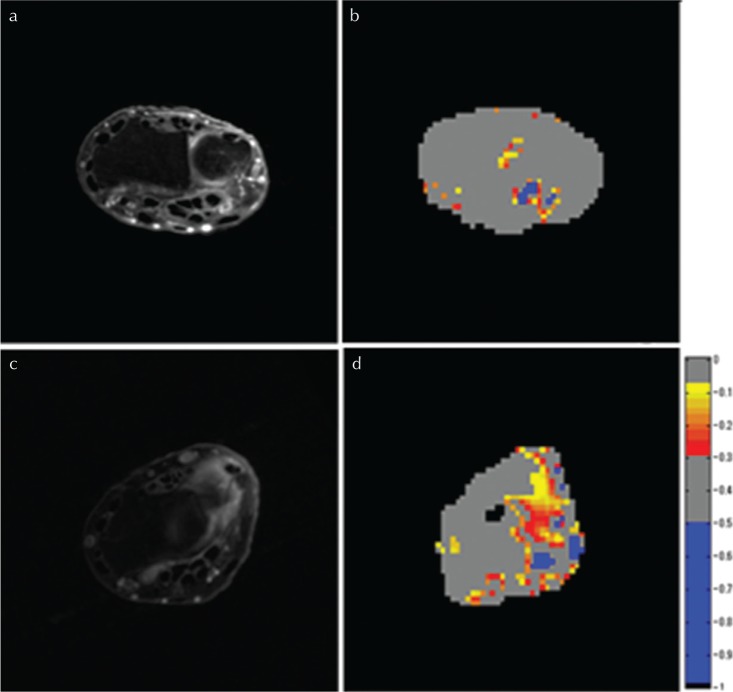
(**a**), (**c**) shows dynamic images for a low (Low-activity group) and a high (high-activity group) degree of synovial proliferation. While, (**b**), (**d**) shows color-coded BFPV maps of (**a**) and (**b**), respectively. The color-coded BFPV maps are calculated by setting threshold values on the gray-scale BFPV map to distinguish pannus from surrounding tissues. Peripheral artery (blue color wash) and pannus (yellow and red color wash) are visualized on each image. BFPV, blood flow pattern variation.

The threshold value indicating pannus was set from −0.2914 to −0.0694 based on the interquartile range of BFPV values. Similarly, the upper limit value indicating peripheral artery was set as −0.5009. Applying these threshold values to the gray-scale BFPV maps, color-coded BFPV maps were calculated as to separate pannus and peripheral artery from surrounding tissue, respectively (Fig. 5). In terms of the presence and extent of pannus formation on dynamic images or, all subjects were divided into two groups: five patients classified in high-activity group showed apparent pannus formation (with a range of the volume of pannus measured by manual outlining from 9576 mm^3^ to 26960 mm^3^) and three patients classified in low-activity group showed no, or a limited extent of hyperemia in pannus (with the volume of pannus measured by manual outlining ranging from 0 mm^3^ to 2554 mm^3^). Regarding the extent of *Pan*_thre_, the Independent *t*-test showed significant difference between the high-activity group and the low-activity group (*P* = 0.023). The high-activity group showed a significantly larger *Pan*_thre_ than the low-activity group.

## Discussion

In this study, the gray-scale/color-coded blood flow pattern variation (BFPV) map was introduced for differentiation of normal and abnormal tissues in patients with RA. The gray-scale BFPV map was calculated by conventional ASL images but obtained with two separate PLD times, and represented the relative variation in blood flow pattern. This study demonstrated that the pannus and the other tissues including the peripheral artery, showed significantly different BFPV values. In addition, the independent *t*-test revealed that the high-activity group showed a significantly larger volume of pannus (*Pan*_thre_) on color-coded BFPV maps than the low-activity group. The color-coded BFPV map was in agreement with contrast-enhanced dynamic images regarding assessment of the degree of RA synovitis. These results indicated that the degree of inflammatory tissue may be assessed noninvasively using the color-coded BFPV map.

In a previous study, conventional ASL perfusion imaging of synovitis in inflammatory arthritis of finger and wrist joints at high-field strength was concluded to be feasible.^1^ They determined a threshold for perfusion values (over 600 ml/100 g/min) to distinguish pannus from vessels, based on perfusion values of macroscopic vascular flow rather than tissue perfusion. However, the authors needed to define multiple thresholds for perfusion values to identify pannus from other tissues and found that the perfusion values of each tissue fluctuate dramatically depending on PLD times. Thus, the method described in the previous study of extracting microvascular needs to determine the appropriate delay time to allow the label to reach the tissue of interest. This is not practical because predicting proper PLD time is not possible.

In the gray-scale BFPV map, because the blood containing labeled spins travels and passes faster through the artery than in the pannus, the average BFPV values of the artery are negative and are smaller than those of pannus. On the other hand, as the labeled arterial spins gradually permeate the other tissues, their average BFPV values tend to be positive. This approach may perform tissue characterization related to the inflow and outflow ratio of the blood containing labeled spins, that is, relative variation in blood-flow pattern or perfusion.

Limitations to this study must be addressed here. First, although we proved that proper BFPV setting is useful in discriminating the voxels of pannus from those of other tissues, we utilized dynamic MRI data in defining the BFPV values of the pannus. Verification is required whether the thresholding of the BFPV is universally applied, which is beyond the scope of this study. Second, as the number of cases included in this study is small, PLD settings used to calculate the gray-scale BFPV map may not be applied to every patient with RA. Also, as selection/combination of PLD times were determined according to our experience, refinements on proper PLD settings may be required. Third, for the quantitative mapping of wrist perfusion, the standard pCASL model for the brain was adopted, which was not validated for the wrist. For example, the efficiency of the pulsed-continuous labeling was estimated at approximately 0.85 based on previous simulations.^9^ Actually, the efficiency of the labeling is reduced further due to the inefficiency of the background suppression inversion pulses affected by the labeled spins.^10^ Therefore, it is considered that pCASL perfusion maps were affected by this constant for quantification. However, the gray-scale/color-coded BFPV map represents the variation in the relative blood-flow pattern and does not totally depend on the absolute perfusion value itself. Considering the successful discrimination of pannus from other tissues, we believe the analysis for the gray-scale/color-coded BFPV map may be feasible in spite of the use of pCASL, which is not dedicated for the wrist. Forth, within the imaging volume, which was set from the distal radio-ulnar joint to the carpometacarpal joints, it was not recognized that average BFPV values of specific tissue change dependent on the distance of slice levels from the labeled plane. However, as the blood flow velocity slows toward the periphery, there is a possibility that the BFPV values of tissue at the region over carpometacarpal joints have different characteristics from this study, and it may not clearly identify pannus formation from artery on color-coded BFPV maps. This should be elucidated in the future study as well as the reproducibility of this method including the manner of ROI placement. Fifth and finally, healthy control subjects did not participate in this study. To compensate this, we divided patients into active and inactive groups for synovitis depending on the degree of pannus volume, and it was possible to show a significant difference between the two groups. These results do not reveal that the subjects who have no inflammatory tissue are seen on color-coded BFPV maps as having little pannus volume.

## Conclusion

In conclusion, the relative variation in the blood-flow pattern, calculated by ASL perfusion imaging obtained with two different PLD times, was introduced and visualized as the gray-scale BFPV map. This study demonstrated a statistically significant difference in the BFPV values among artery, pannus, and surrounding tissue due to the advantage of precise tissue characterization related to the blood-flow pattern of the labeled arterial spins. Moreover, the color-coded BFPV map could accurately distinguish pannus from other tissues including the peripheral artery. These results suggest that this approach may be capable of identifying pannus quantitatively and noninvasively without the need to determine the appropriate PLD time to allow the label to reach the tissue of interest.
